# Waste separation—Who cares? Organizational climate and supervisor support’s role in promoting pro-environmental behaviors in the workplace

**DOI:** 10.3389/fpsyg.2022.1082155

**Published:** 2022-12-22

**Authors:** Adriana Costa, Carla Mouro, Ana Patrícia Duarte

**Affiliations:** ^1^Department of Psychology, Iscte-Instituto Universitário de Lisboa, Lisbon, Portugal; ^2^Centro de Investigação e Intervenção Social, Iscte-Instituto Universitário de Lisboa, Lisbon, Portugal; ^3^Business Research Unit, Iscte-Instituto Universitário de Lisboa, Lisbon, Portugal

**Keywords:** pro-environmental behavior, waste separation, recycling, theory of planned behavior, organizational climate, supervisor support

## Abstract

Corporate environmental sustainability is currently a major goal of many businesses. This study’s main objectives were to examine the interactive role of green organizational climate and supervisor support in predicting pro-environmental behaviors (PEBs) at work, namely paper and plastic waste separation, and to test the mediating role of individual-level variables of the Theory of Planned Behavior (TPB) in this relationship. The research specifically tested the attitude, subjective norms, and perceived behavioral control’s mediation of the relationship between green organizational climate and self-reported waste separation, and whether supervisor support moderates the relationship between green climate and TBP variables. Data for this cross-sectional study were collected with an online survey of 311 workers and multiple regression analyses, with the macro Process, were performed to test the hypotheses. The findings confirm the TPB variables’ mediating effect. Perceived green climate is positively related to employees’ attitudes, subjective norms, and perceived behavioral control regarding waste separation, which in turn are connected to higher reported levels of paper and plastic separation. The workers’ perception of supervisor support moderates the relationship between green climate and subjective norms in favor of paper separation. These findings indicate that green climate is less strongly linked to subjective norms when supervisor support is perceived as stronger than when it is seen as weaker. In conclusion, a green organizational climate plays a determining role in workers’ separation of waste at work as it fosters individual motives to perform these behaviors. Moreover, supervisors can provide their workers with social norms and inspire them to support environmental sustainability practices. Thus, as part of an overall transition strategy to achieve sustainability, organizations need to invest in green policies and practices and incentivize supervisors to encourage PEBs and capitalize on their close links to subordinates to foster sustainable norms.

## 1. Introduction

The 2030 Agenda for Sustainable Development lists as one of its goals (i.e., Sustainable Development Goal 12) to ensure sustainable consumption and production patterns (United Nations, 2015). This objective states that waste production must be substantially reduced by 2030 *via* the prevention, reduction, recycling, and reuse of materials. Recycling recovers secondary raw materials, so it decreases greenhouse gas emissions, which means that encouraging waste separation in the workplace is essential for this goal to be achieved.

The issue of pro-environmental behavior (PEBs) at work has become increasingly important as more and more organizations have implemented corporate social responsibility (CSR) strategies (Young and Tilley, 2006). For most companies, CSR and sustainability strategies can contribute to improving environmental performance, especially when employees help develop these strategies (Boiral, 2005; Michailide and Lipsett, 2013). The present study was conducted in response to a specific organization’s request for assistance with an intervention proposal focused on increasing PEBs on its premises, in particular, the separation of paper and plastic waste. This request presented a unique opportunity to examine barriers and facilitators of waste separation in a specific organizational context.

The decision to recycle waste in organizational contexts raises complex issues as many factors are involved at the individual (e.g., perceived social pressure and attitudes) and organizational level (e.g., supervisor support; Tudor et al., 2008; Yuriev et al., 2018). At the individual level, Ajzen’s (1991) theory of planned behavior (TPB) has been widely used in studies of the adoption of PEBs (Steg and Vlek, 2009; Yuriev et al., 2020a). This theory suggests behaviors are fostered by intentions driven by a combination of attitude (i.e., how much an individual values the action in question), subjective norms (i.e., whether an individual perceives that other people think he or she should act that way), and perceived behavioral control (i.e., whether an individual believes he or she has the means to perform the action). The TPB explains a significant amount of variability in the adoption of PEBs, including waste separation (Greaves et al., 2013; Botetzagias et al., 2015). Nevertheless, a recent literature review highlights that the use of TPB is still incipient in workplace studies (Yuriev et al., 2020b), indicating that how organizational decisions affect these variables and how these variables affect employees’ PEBs is an overlooked area of research.

In addition to individual factors, organizational-level variables such as organizational climate and supervisor support can encourage the adoption of PEBs. A green organizational climate refers to employees’ shared perceptions of their firm’s environmental policies, procedures, and practices (Norton et al., 2014). Workers may believe that their employer has formally adopted green policies if organizational procedures support sustainable actions and daily workplace practices reflect the company’s pro-environmental goals and values (Norton et al., 2014). If staff members consider their employer to be socially responsible, they are more likely to engage in organizational citizenship behaviors, of which PEBs are a part (Rupp et al., 2006; Hansen et al., 2011; Temminck et al., 2015; Mouro and Duarte, 2021). Addressing a lacune in the literature regarding PEBs in the workplace (Yuriev et al., 2020b), this research examines how perceptions of a green organizational climate relate to TPB variables, considering these as mediators of the climate-behavior relationship.

Supervisor support is defined as individuals’ belief that their managers value specific behaviors and offer work-related assistance to help in their performance (Susskind et al., 2003), thereby inducing the feeling that their leader wants to encourage these practices (Ramus, 2001). Workers’ PEBs and initiatives are sensitive to the observed daily behavior of their direct supervisors (Daily et al., 2009; Wesselink et al., 2017; Priyankara et al., 2018). Workers can be expected to engage more often in PEBs when they feel their supervisors actively engage in or favor actions that contribute to their organization’s environmental goals. However, this is not always the case; according to Paillé and Francoeur (2022), supervisor support appears to be a conditional factor that may or may not strengthen the organizations’ investment in environmental policies and practices. Another novelty of this research is, therefore, trying to clarify conditions in which supervisor support contributes to workers’ adherence to PEBs.

The current study thus sought to examine the interactive effect of individual-level variables (i.e., the TPB), and organizational-level variables’ (i.e., supervisor support and green organizational climate) on waste separation in order to increase this behavior in the workplace. More specifically, this research explored TPB variables’ (i.e., attitude, subjective norms, and perceived behavioral control) mediation of the relationship between green organizational climate and separation of paper and plastic waste. In addition, it was tested whether supervisor support moderates the link between green organizational climate and TPB variables. The results add to the existing literature by clarifying whether a supportive organizational climate and supervisor encouragement contribute—jointly or independently—to employees feeling personally committed to and able to engage in PEBs at work.

## 2. Theoretical background and hypotheses development

### 2.1. Theoretical background

#### 2.1.1. Pro-environmental behaviors at work

Pro-environmental behaviors can be defined as actions that intentionally seek to reduce people’s negative impact on the environment (Stern, 2000; Boiral et al., 2015a). They can be defined by their impact, that is, by the extent to which they alter the environment or modify ecosystems, the biosphere, and climates’ structure and dynamics (Stern, 2000). This type of behavior can directly reduce pressures on the environment, such as when individuals separate waste or clean urban and/or forest parks, or have an indirect effect by modifying the context in which decisions are made that affect environmental trends (e.g., environmental tax policies; Stern, 2000).

This type of behavior at work has the potential to contribute significantly to mitigating organizations’ negative environmental effects (Ones and Dilchert, 2012; Robertson and Barling, 2013; Blok et al., 2015; Tsai et al., 2016). Most studies have, however, focused on PEBs at home (Ruepert et al., 2016; Whitmarsh et al., 2018; Qalati et al., 2022), so gaps still exist in the literature on these behaviors in the workplace. Researchers have only recently begun to try to understand the individual and organizational factors fostering workers’ adoption of PEBs, as discussed in the next subsections.

#### 2.1.2. Organizational climate promoting pro-environmental behaviors at work

Organizational climate can be defined as the companies’ existing atmosphere, which emerges from employees’ daily practices and procedures, and which is intrinsically connected to supervisors’ actions and the behaviors they reward (Schneider et al., 2013). To study organizational climate, researchers must examine individual factors related to workers’ values and needs, but also the organizational policies, norms, and codes (Argyris, 1958).

A green organizational climate arises when firms seek to develop and improve policies and methods that engage their workers more fully in PEBs. This kind of climate can be defined as employees’ shared perceptions of their organization’s environmental policies, procedures, and practices (Norton et al., 2012; Chou, 2014). These perceptions are formed when companies formally adopt environmentally friendly policies, different departments adopt procedures and practices that support sustainable actions, and day-to-day routines reflect internal pro-environmental values and objectives (Norton et al., 2012). Studying this construct’s application in organizations can provide more information about determinants of environmentally sustainable performance, which in turn contributes to a fuller understanding of how employees’ PEBs can be generated and promoted in the workplace (Norton et al., 2012).

Previous research has confirmed that a positive relationship exists between green organizational climate and PEBs at work (Wesselink et al., 2017; Zientara and Zamojska, 2018), especially in organizations that adopt sustainability policies (Norton et al., 2014) and green human resource management practices (Dumont et al., 2017). Hicklenton et al. (2019) further found that workers who report that their company is committed to implementing sustainable strategies and procedures register higher levels of motivation to engage in PEBs. By including environmental issues in their organizational mission, firms increase the likelihood that employees will engage in PEBs, informally encourage managers to support these actions more fully, and establish their pro-environmental reputation in the public’s eyes (Zibarras and Ballinger, 2011).

#### 2.1.3. Theory of planned behavior promoting pro-environmental behaviors at work

The TPB (Ajzen, 1991) was developed based on the theory of reasoned action (Fishbein and Ajzen, 1975). The TPB postulates that behaviors are the result of individuals’ intention to perform them and that intentions have three determinants. The first is the attitude or a positive or negative assessment of a particular action (e.g., I am very much in favor of separating waste in my organization.). The second is subjective norms or perceptions of social pressures to perform a certain behavior (e.g., My colleagues think it is important that I separate paper waste for recycling.). The last determinant is perceived behavioral control or a perception of one’s ability to perform a particular action (e.g., I think that the containers for waste separation are in an accessible place.).

In general, the more favorable an individual’s attitudes and subjective norms are toward a given behavior and the greater their perceived behavioral control, the stronger their intention to perform that behavior will be. However, the relative importance of attitude, subjective norms, and perceived behavioral control varies according to the behaviors and situations under analysis. In some situations, only attitudes may have a significant impact on intentions. In others, both attitudes and perceived behavioral control can sufficiently explain intentions, while in others all three predictors make significant independent contributions (Ajzen, 1991).

The TPB has been applied in previous studies to clarify the factors determining PEBs in the workplace. For example, Greaves et al. (2013), Norton et al. (2015), and Wesselink et al. (2017) report that attitude and subjective norms are the individual factors that most affect PEBs at work. In contrast, Razak and Sabri (2019), Canova and Manganelli (2020), and Khalid et al. (2022) assert that behavioral control is the strongest predictor of employees’ PEBs intentions in organizations. The TPB has also specifically been used to explain intentions to perform waste separation at work in prior research. In some studies, a favorable attitude was the strongest predictor (Laudenslager et al., 2004; Tudor et al., 2007), but Blok et al. (2015), Botetzagias et al. (2015), and Ofstad et al. (2017) found instead that perceived behavioral control was the most significant factor contributing for this type of behavior.

The existing literature thus supports the conclusion that no single predictive pattern is associated with the TPB because the strongest determinants appear to vary according to the research context in question. Additional investigations are needed to identify the most important predictors of waste separation and explore which factors can alter TPB variables’ predictive power. More concretely, PEBs in the workplace may be influenced by factors that do not exist at home, such as organizational values or supervisors and colleagues’ encouragement (Blok et al., 2015; Yuriev et al., 2018; Mouro and Duarte, 2021).

Perceptions of a strong green organizational climate can generate meaningful reflection about pro-environmental practices (Afsar and Umrani, 2020) and lead to clearer personal norms (Chou, 2014; Zientara and Zamojska, 2018; Mouro and Duarte, 2021; Sharpe et al., 2022). If organizations invest strongly enough in a green organizational climate, employees will most likely see their company’s values and actions as environmentally friendly, and this perception will contribute to these workers’ positive personal beliefs, intrinsic motivation and norms about PEBs at work (Chou, 2014; Mouro and Duarte, 2021; Sharpe et al., 2022). Given enough time, workers’ behavior will be guided by their personal commitment to environmental sustainability. Staff members may bring positive environmental attitudes to their workplace, but an organizational climate that supports environmental protection can reinforce these attitudes among workers (Russell and Griffiths, 2008).

Perceived behavioral control is also related to a green organizational climate (Lülfs and Hahn, 2013). Employees’ perception of their ability to perform their PEBs is higher when the employer is thought to be an environmentally friendly organization, and these workers will be more inclined to engage in these behaviors (Afsar et al., 2020).

Subjective norms can also be influenced by a green organizational climate. Afsar and Umrani (2020) report that perceived social responsibility is significantly linked with higher pro-environmental advocacy toward coworkers. Fellow employees’ green advocacy refers to the extent to which coworkers openly discuss environmental problems and possible solutions, share relevant knowledge, inform others about ecological issues, and communicate their ideas about ways to improve the environment in order to encourage others to engage in PEBs. A green organizational climate can influence workers’ subjective norms so that the stronger the employees’ perception that their organization supports environmental protection, the more intense will be their awareness of social pressure from colleagues and other staff members to join in PEBs. Given the extant literature on this topic, the present study sought to establish whether the positive relationship between green organizational climate and PEBs is mediated by TPB variables.

#### 2.1.4. Supervisor support and pro-environmental behaviors at work

Supervisors’ conduct is especially important to the formation of an organizational climate, which means that managers should not only provide reasons for specific organizational initiatives to their subordinates but also set an example to inspire them (Zientara and Zamojska, 2018). The literature on PEBs at work refers to two types of supervisor support. The first is general encouragement or the degree to which managers value their employees’ contributions and care about their well-being (Eisenberger et al., 1986). The second is support for environmental sustainability, which can be defined as when supervisors attach importance and offer assistance related to PEBs (Cantor et al., 2015). Notably, Paillé et al. (2013) found that general encouragement has a negative impact on workers’ PEBs and called for more research to clarify this relationship more fully. The cited authors further suggested that supervisors’ specific support for environmental sustainability should be explored instead of general support as a predictor of PEBs.

Supervisors’ actions are significant determinants of workers’ attitudes (Ramus, 2001). Supervisor support for PEBs can be a fundamental stimulus for workers less concerned about environmental sustainability in work contexts (Paillé et al., 2019). According to Norton et al. (2015), a lack of supervisor support was found to be an important obstacle to pro-environmental initiatives in a quarter of the studies analyzed. Employees who felt that their supervisors encourage them are overall more likely to engage in pro-environmental initiatives and behaviors (Ramus, 2002; Boiral et al., 2015b; Wesselink et al., 2017), thereby providing the basis for greater trust to be built within organizations (Whitener et al., 1998). Supervisors can transmit and demonstrate their company’s environmental policies, procedures, and practices and strengthen their subordinates’ perception that their organization is committed to environmental sustainability, including genuinely valuing PEBs (Daily et al., 2009; Cantor et al., 2012; Robertson and Carleton, 2018). Supervisors thus provide workers with social norms because they transmit to them other staff members’ expectations within their organization (Daily et al., 2009).

Blok et al. (2015) suggest that supervisors should focus on reinforcing employees’ attitudes, social norms, and perceived behavioral control in connection to PEBs. From a self-determination theory perspective (Deci and Ryan, 2008), supervisor support related to environmental issues strengthens workers’ feeling of competence and autonomy with regard to PEBs, thereby increasing their perceived behavioral control (Priyankara et al., 2018). Supervisors who lead by example and encourage employees to engage in environmental issues voluntarily and spontaneously can increase their subordinates’ perceived control over pro-environmental initiatives and behaviors (Boiral et al., 2015b), especially for individuals with personal ecological beliefs (Raineri and Paillé, 2016).

However, supervisor support’s role in workers’ engagement in PEBs is still unclear as this type of encouragement’s effect appears to be dependent on the type of support evaluated and the organizational initiatives implemented, that is, the presence of a green organizational climate. Paillé and Francoeur (2022) observe that organizational climate and supervisor support may not have additive effects. Organizational climate’s impact may instead depend on the level of supervisor support perceived by employees. The present study thus explored supervisor support’s moderating effect on the relationship between green organizational climate and TPB variables, positing that the association between green organizational climate and individual attitude, subjective norms, and perceived control is stronger when supervisor support is weak. When supervisors’ support is strong, green organizational climate’s direct role in individuals’ perceptions will be smaller.

### 2.2. Hypotheses development

As mentioned, although TPB variables showed consistency in predicting waste separation in the domestic context, there is limited research on their role in work contexts and on how they may be affected by organizational level variables. This study, therefore, focused on green organizational climate’s role in predicting waste separation at work, and tested the mediating effect of attitudes, subjective norms, and perceived behavioral control on this relationship. In addition, this research also focused on whether supervisor support moderates the relationship between green organizational climate and the three TPB variables. Inconsistent findings on the role of supervisor support justify exploring whether its impact on PEBs is achieved through the modulation of workers’ motives for engaging in such behaviors. The behaviors of paper and plastic separation were analyzed separately to establish whether the factors under study have different weights for each type of waste.

The following hypotheses were formulated and tested:

*H1*: Green organizational climate is positively related to (paper and plastic) waste separation behaviors.

*H2*: The relationship between green organizational climate and (paper and plastic) waste separation is mediated by TPB variables.

*H3*: Perceived supervisor support moderates the relationship between green organizational climate and TPB variables. The association between green organizational climate and TPB variables is stronger when supervisor support is weaker than strong.

**Figure 1:**
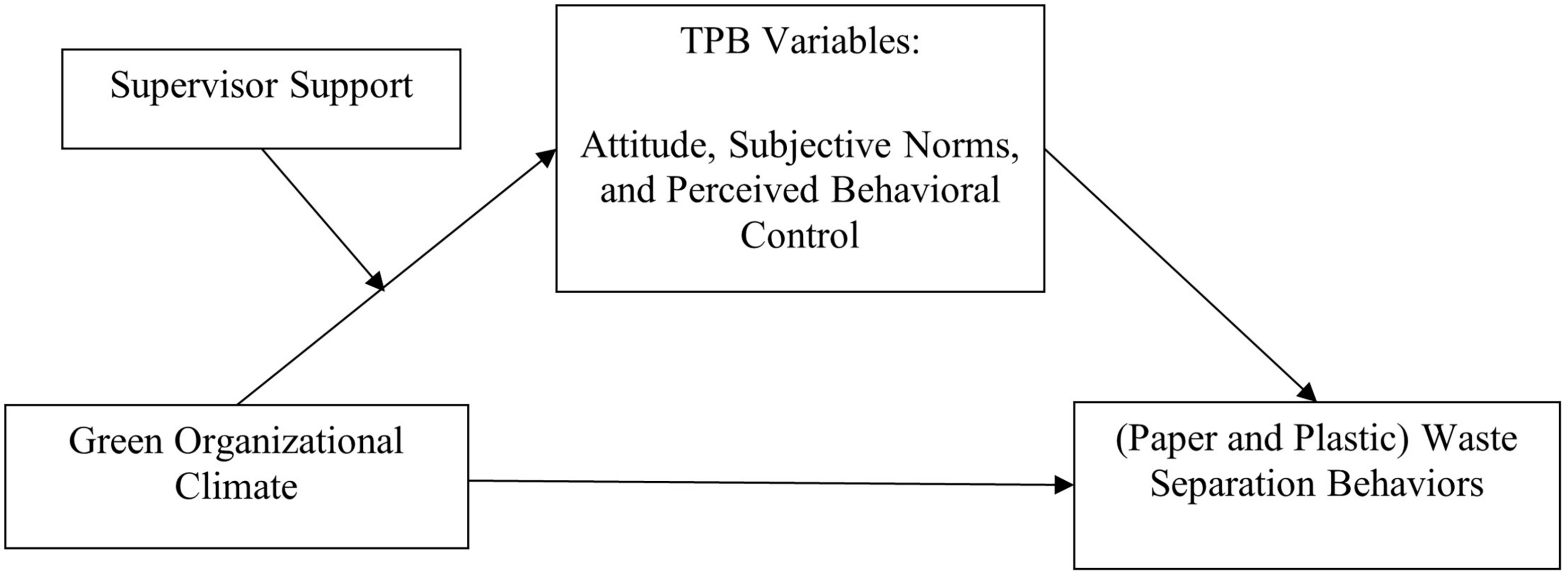
Research model.

The research model is presented in Figure 1.

## 3. Materials and methods

### 3.1. Research context

This study sought to respond to a tertiary sector organization’s request for assistance with how to increase the separation of waste (i.e., paper and plastic) in its central building. This organization had set sustainable development as one of its strategic objectives. The headquarters where the central services are located housed more than 1,400 employees. The research was conducted in March 2020, immediately before the first lockdown was imposed because of the COVID-19 pandemic.

### 3.2. Participants

A survey was administered online, and 311 valid complete responses were collected. The participants were between 23 and 67 years old (*M* = 46.29; SD = 9.58), and 60.1% were female. Regarding the level of schooling, 45.0% had a higher education degree, while 24.1% had a high school degree. The respondents had worked for the organization on average for 20 years (SD = 12.07; maximum = 45; minimum = 0.25). Most participants had a permanent employment contract (96.1%) and did not have a supervisor position (74.9%).

### 3.3. Procedures and instruments

The study was quantitative, correlational, and cross-sectional, based on a survey of workers. An online questionnaire was used for data collection because this constitutes a cost-effective, efficient way to obtain data for statistical analysis from large samples, being a widely employed data-collection strategy among the research community (Hill and Hill, 2005; Qalati et al., 2022). A non-probabilistic sampling procedure was used to collect the data. The questionnaire was developed in the Qualtrics online platform, and the online link was sent *via* a message issued by the Department of Internal Communication to employees’ institutional e-mail with the subject “Survey of waste treatment in the central building.” The data were collected over 2 weeks. Only the research team had access to the responses recorded on the platform.

The questionnaire consisted of a first page presenting the study’s aims and an informed consent form. The respondents were told that participation was voluntary, anonymous, and confidential; the data would be statistically analyzed only by the team; and no responses would be processed or reported individually. The items assessing the variables under study were presented in the following order: green organizational climate, perceived supervisor support, TPB variables (i.e., randomly presented for paper and plastic), and sociodemographic questions (i.e., age, gender, education, seniority, work contract, and supervisor position). The scales used to collect the data are presented below and a complete list of items used can be found in the Appendix.

#### 3.3.1. Predictor variable: Green organizational climate

Nine items based on Norton et al. (2014), Ramus (2002), and Zibarras and Ballinger’s (2011) work were evaluated on a 5-point response scale (1 = “Strongly disagree”; 5 = “Totally agree”), including “my organization … provides appropriate containers for paper/plastic separation.” This scale has a high internal consistency [Cronbach’s alpha (*α*) = 0.90].

#### 3.3.2. Criterion variable: Waste separation behavior

The following item was included to evaluate how much employees were separating waste (Tonglet et al., 2004): “I have separated paper/plastic waste for recycling for the last 4 weeks.” A 5-point Likert response scale was used (1 = “I totally disagree.”; 5 = “I totally agree”).

#### 3.3.3. Mediating variables

##### 3.3.3.1. Attitude

This variable’s items were taken from Tonglet et al.’s (2004) study and evaluated on a 7-point semantic differential scale (e.g., “To separate paper/plastic for recycling is something … 1 = positive; 7 = negative”/). Both the measures for paper separation (*α* = 0.65) and plastic separation (*α* = 0.69) present adequate internal consistency.

##### 3.3.3.2. Subjective norms

The three items assessing this variable were adapted from Sidique et al.’s (2010) scale (e.g., “My direct supervisors/colleagues/coworkers expect me to separate paper/plastic waste.”). A 5-point Likert response scale was used (1 = “I totally disagree”; 5 = “I totally agree”). The indicators for paper separation (*α* = 0.92) and plastic separation (*α* = 0.93) have a very good internal consistency.

##### 3.3.3.3. Perceived behavioral control

To evaluate this variable, five items were adapted from Tonglet et al.’s (2004) work (e.g., “I know where to take paper/plastic for recycling.”). A 5-point Likert response scale was utilized (1 = “I totally disagree”; 5 = “I totally agree”). The measures for paper (*α* = 0.78) and plastic separation (*α* = 0.74) have a good internal consistency.

#### 3.3.4. Moderating variable: Supervisor support

Three items were developed based on Priyankara et al. (2018), Ramus (2001), and Susskind et al.’s (2003) research. A sample item is “My direct supervisor encourages me to incorporate environmentally friendly practices into my work.” The responses were given on a 5-point scale (1 = “Never”; 5 = “Almost always or always”). The measure has a very good internal consistency (*α* = 0.93).

### 3.4. Common method variance

Given the use of a single source at a single moment in time to collect data, common method variance (CMV) could weaken the results’ validity (Podsakoff et al., 2003), despite this being unusual (Bozionelos and Simmering, 2022). As suggested by Podsakoff et al. (2003) different rating scales were used, the respondents’ anonymity and their answers’ confidentiality were guaranteed, and evaluation apprehension was diminished by assuring respondents of the absence of no right or wrong answers. As well, Harman’s test was applied to assure data robustness to CMV, which uses exploratory factor analysis without rotation to determine the number of factors needed to explain the relevant variables’ variance. The results reveal that the first factor only accounts for 29.30% of the total variance (66.24%), indicating the absence of any serious CMV (Kaiser-Meyer-Olkin test = 0.87; Bartlett’s test = 9326.43; *p* < 0.001).

## 4. Results

The data were analyzed using IBM’s SPSS Statistics 26 software, and the mediation and moderation models were tested with the assistance of Hayes’s (2018) Process macro version 3.5. The results for the descriptive statistics and correlations are presented below first for paper separation and second for plastic separation. The outcomes responding to the hypotheses are then given in the same order.

Table 1 presents the variables’ correlations and *M*, SD, and reliability values for paper separation. The participants’ responses regarding organizational variables indicate that a moderately strong green organizational climate (*M* = 3.28; SD = 0.80) is present and that their direct supervisors provide mild support for PEBs at work (*M* = 2.74; SD = 1.13). Green organizational climate has a significant positive association with supervisor support (Spearman’s *rho* = 0.45; *p* < 0.01; see Table 1).

**Table 1:** Descriptive statistics, correlations, and reliabilities for paper separation.

Variables	*M*	SD	1	2	3	4	5	6	7
1. Age	46.29	9.58	-						
2. Seniority	20.20	12.04	0.85^**^	-					
3. Green Organizational Climate	3.28	0.81	0.15^*^	0.14^*^	(0.90)				
4. Attitude	6.51	0.87	0.00	−0.04	0.19^**^	(0.65)			
5. Subjective norms	3.38	0.98	0.09	0.09	0.52^**^	0.18^**^	(0.92)		
6. Perceived behavioral control	3.90	0.80	0.14^*^	0.16^**^	0.43^**^	0.26^**^	0.35^**^	(0.78)	
7. Supervisor support	2.73	1.13	−0.02	−0.01	0.45^**^	0.14^*^	0.59^**^	0.17^**^	(0.93)
8. Paper separation	4.28	1.07	0.21^**^	0.19^**^	0.27^**^	0.27^**^	0.29^**^	0.52^**^	0.09

*M* = mean; SD = standard deviation. ^*^p < 0.05; ^**^p < 0.01. Cronbach’s alpha coefficients in parentheses.

On average, the workers surveyed report that they have recycled paper in their workplace many times (*M* = 4.28; SD = 1.07). They also have an extremely positive attitude toward paper waste separation (*M* = 6.51; SD = 0.87) and feel moderate social pressure to separate paper waste (*M* = 3.38; SD = 0.97) and substantial control over paper separation (*M* = 3.91; SD = 0.78). Regarding organizational variables, the green organizational climate has a significant positive link with paper separation behaviors (*rho* = 0.27; *p* < 0.01). Perceived supervisor support does not present a significant correlation with the same behaviors [*rho* = 0.09; not statistically significant (n.s.)]. Since supervisor support is a moderating variable in this study, this missing correlation did not prevent further analysis of the relevant hypothesis.

Perceptions of a stronger green organizational climate are associated with more positive attitudes about (*rho* = 0.19; *p* < 0.01), perceived social pressure to engage in (*rho* = 0.52; *p* < 0.01), and perceived control over (*rho* = 0.57; *p* < 0.01) paper separation (see Table 1 above). TPB variables are also positively and significantly correlated with paper recycling behaviors (*rho* attitude = 0.27; *p* < 0.01; *rho* subjective norm = 0.29; *p* < 0.01; *rho* perceived control = 0.52; *p* < 0.01). Perceived supervisor support and TPB variables are also positively and significantly linked (see Table 1 above).

Age (*rho* = 0.21; *p* < 0.01) and seniority (*rho* = 0.19; *p* < 0.01) are the only sociodemographic variables significantly correlated with paper separation behaviors. Because of these factors’ strong association (*rho* = 0.85; *p* < 0.01), only seniority was included in subsequent analyses.

Table 2 shows the variables’ descriptive statistics and correlations for plastic recycling. On average, these employees recall separating plastic in the workplace many times (*M* = 4.14; SD = 1.11). In addition, they have an extremely positive attitude toward plastic waste separation (*M* = 6.35; SD = 0.96), feel moderate social pressure to separate plastic waste (*M* = 3.33; SD = 0.99), and report strong control over when and how they recycle plastic (*M* = 3.71; SD = 0.81).

**Table 2:** Descriptive statistics, correlations, and reliabilities for plastic separation.

Variables	*M*	SD	1	2	3	4	5	6	7
1. Age	46.29	9.58	-						
2. Seniority	20.20	12.04	0.85^**^	-					
3. Green organizational climate	3.28	0.81	0.15^*^	0.14^*^	(0.90)				
4. Attitude	6.35	0.96	0.01	−0.05	0.14^*^	(0.69)			
5. Subjective norms	3.33	0.99	0.07	0.06	0.52^**^	0.18^**^	(0.93)		
6. Perceived behavioral control	3.71	0.81	0.06	0.06	0.45^**^	0.28^**^	0.38^**^	(0.74)	
7. Supervisor support	2.73	1.13	−0.02	−0.01	0.45^**^	0.09	0.59^**^	0.21^**^	(0.93)
8. Plastic separation	4.14	1.11	0.18^**^	0.15^**^	0.24^**^	0.21^**^	0.27^**^	0.49^**^	0.07

*M* = mean; SD = standard deviation. ^*^p < 0.05; ^**^p < 0.01. Cronbach’s alpha coefficients in parentheses.

Analyses of the organizational variables revealed that green organizational climate has a significant positive association with plastic separation behaviors (*rho* = 0.24; *p* < 0.01). Supervisor support has a positive but non-significant correlation with these recycling practices (*rho* = 0.07; n.s.). Green organizational climate, in turn, is both positively and significantly correlated with all TPB variables: attitude (*rho* = 0.14; *p* < 0.05), subjective norms (*rho* = 0.52; *p* < 0.01), and perceived control (*rho* = 0.45; *p* < 0.01). TPB variables also have a significant positive link with plastic separation habits: attitude (*rho* = 0.21; *p* < 0.01), subjective norms (*rho* = 0.27; *p* < 0.01), and perceived control (*rho* = 0.49; *p* < 0.01).

Supervisor support is not significantly correlated with attitude (*rho* = 0.09; *p* > 0.05) but is associated with stronger perceived social pressure to engage in (*rho* = 0.59; *p* < 0.01) and control over plastic separation (*rho* = 0.21; *p* < 0.01; see Table 2). Age (*rho* = 0.18; *p* < 0.01) and seniority (*rho* = 0.15; *p* < 0.01) are also correlated with plastic recycling, yet, as was decided for paper separation, only seniority was controlled for in subsequent analyses.

*T*-tests were run with paired samples to compare the variables associated with paper and plastic separation. The results show that attitude [*t* (310) = 1.58; n.s.] and subjective norms have similar levels for both paper and plastic. Perceived behavioral control, however, is greater for paper than for plastic recycling [*t* (310) = 6.11; *p* < 0.001]. The level of reported paper separation is higher than that of plastic separation [*t* (310) = 2.89; *p* < 0.01].

### 4.1. Testing of paper separation model

Prior to hypotheses testing, tolerance and variance inflation factor (VIF) values were examined for assessing multicollinearity. The results produced tolerance values between 0.58 and 0.94 and VIF values between 1.07 and 1.72, indicating that multicollinearity was not present in the data (Cohen et al., 2003).

The first hypothesis proposed that a green organizational climate is positively associated with waste separation behaviors. The results indicate that green organizational climate has a significant positive total effect on paper recycling [non-standardized regression coefficient (*B*) = 0.41; *p* < 0.001]. Thus, the stronger the workers’ perception of a green organizational climate at work, the more likely they are to report frequently separating paper waste. *H*1 is thus corroborated.

The second hypothesis proposed that TPB variables mediate the aforementioned relationship. The results reveal that green organizational climate has a significant positive connection to attitude (*B* = 0.23; *p* < 0.01), subjective norms (*B* = 0.65; *p* < 0.001), and perceived behavioral control (*B* = 0.46; *p* < 0.001). These variables are also positively and significantly associated with paper separation behaviors as follows: attitude (*B* = 0.16; *p* < 0.05), subjective norms (*B* = 0.18; *p* < 0.001), and perceived behavioral control (*B* = 0.53; *p* < 0.001). In addition, the three variables’ indirect effect is positive and significant, which provides support for the TPB variables’ mediation: attitude [*B* = 0.04; 95% confidence interval (CI) (0.01, 0.08)], subjective norms [*B* = 0.12; 95% CI (0.02, 0.23)], and perceived behavioral control [*B* = 0.24; 95% CI (0.14, 0.37)]. These results suggest that green organizational climate affects paper recycling behaviors through TPB variables, providing empirical support for *H*2. Green organizational climate is no longer significant in the presence of the mediating variables (*B* = 0.01; *p* > 0.05), so a complete mediation is present. The resulting model explains 32% of the variation in paper separation behaviors [*F* (5, 305) = 29.25; *p* < 0.001] (see Table 3).

**Table 3:** Total, direct, and indirect effects for paper separation.

Variables	Attitude		Subjective norms		Perceived behavioral control		Paper separation behavior	
	*B*	SE	*B*	SE	*B*	SE	*B*	SE
Total effect								
Constant							2.68^***^	0.24
Green Organizational Climate							0.41^***^	0.07
Seniority							0.01^*^	0.00
Direct effect								
Constant	5.89^***^	0.21	1.20^***^	0.20	2.27^***^	0.17	0.31	0.42
Green Organizational Climate	0.23^***^	0.06	0.65^***^	0.06	0.46^***^	0.05	0.01	0.08
Attitude							0.16^*^	0.06
Subjective Norms							0.18^***^	0.06
Perceived Control							0.53^***^	0.07
Seniority	−0.01	0.00	0.00	0.00	0.01^*^	0.00	0.01^*^	0.00
Indirect effect								
	0.04^*^	0.02	0.12^*^	0.05	0.24^*^	0.06		
	95% bootstrap CI							
	[0.01, 0.08]		[0.02, 0.23]		[0.14, 0.37]			

^*^p < 0.05; ^**^p < 0.01; ^***^p < 0.001. *B*, non-standardized regression coefficients; SE, standard error; CI, confidence interval.

*H*3 posited that supervisor support moderates the links between green organizational climate and TPB variables. The results verify a significant moderation of the relationship between green organizational climate and subjective norms for paper separation. The corresponding interaction effect presents a significant negative value (*B* = −0.11; *p* < 0.001), which indicates that the more strongly supervisors support these behaviors, the weaker the association becomes between green organizational climate and subjective norms (see Table 4). Green organizational climate’s indirect effect on paper separation *via* subjective norms implies the existence of significant moderated mediation [*B* = −0.02; 95% CI (−0.05, −0.00)], thereby confirming that these factors’ interactions are more significant for those who have weak supervisor support for paper recycling behaviors.

**Table 4:** Moderation and mediated moderation for paper separation.

Variables	Attitude		Subjective norms		Perceived behavioral control	
	*B*	SE	*B*	SE	*B*	SE
Constant	6.68^***^	0.10	3.32^***^	0.08	3.75^***^	0.08
Green organizational climate	0.20^***^	0.07	0.38^***^	0.06	0.46^***^	0.06
Supervisor support	0.04	0.17	0.40^***^	0.04	0.00	0.04
Seniority	−0.01	0.00	0.01	0.00	0.01	0.00
Interaction effect [GreenClimate*SupervisorSupport]	−0.07	0.05	−0.11^**^	0.04	0.01	0.04
*R*^2^ =	0.05^***^		0.47^***^		0.24^***^	
*F* (4, 306) =	4.43		66.57		24.67	
	Moderated mediation index					
	−0.01		−0.02		0.01	
	95% CI					
	[−0.03, 0.00]		[−0.05, −0.00]		[−0.05, 0.06]	

^*^p < 0.05; ^**^p < 0.01; ^***^p < 0.001. *B*, non-standardized regression coefficients; SE, standard error; CI, confidence interval.

**Figure 2:**
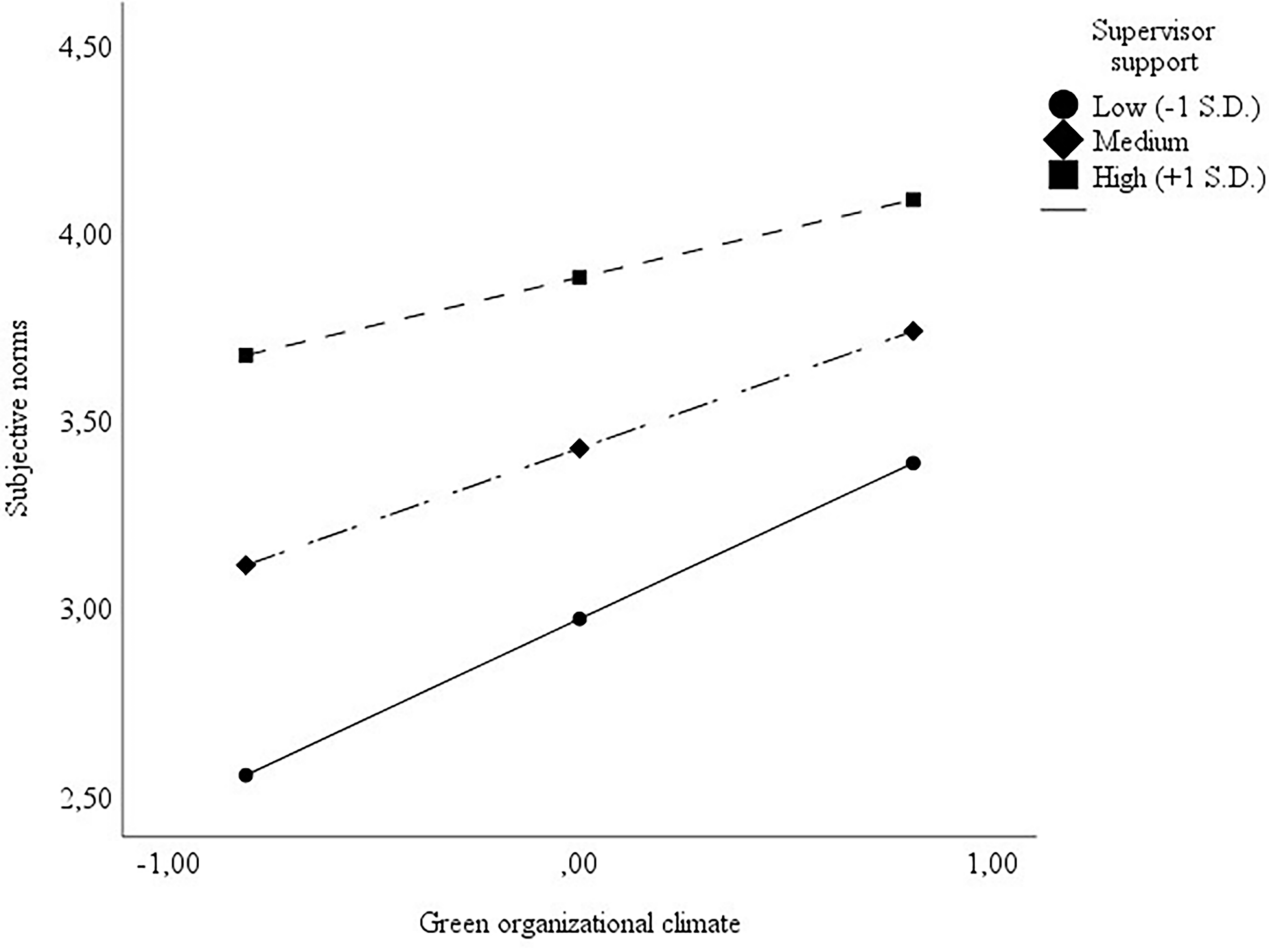
Supervisor support’s moderation of the relationship between green organizational climate and subjective norms (for paper separation).

Figure 2 shows the slopes for three levels of supervisor support: strong or an SD above the mean (*B* = 0.26; *p* < 0.001), medium (*B* = 0.38; *p* < 0.001), and weak or an SD below the mean (*B* = 0.51; *p* < 0.001). These results support the conclusion that supervisor support moderates the relationship between green organizational climate and subjective norms for paper separation, thereby partially confirming *H*3.

The analyses produced no evidence of supervisor support’s moderation of the connection between green organizational climate and attitude (*B* = −0.07; n.s.) or between green organizational climate and perceived control (*B* = 0.01; n.s.). Thus, *H*3 for these TPB variables is not empirically supported (see Table 4 above).

### 4.2. Testing of plastic separation model

Once more, prior to hypotheses testing, tolerance and variance inflation factor (VIF) values were determined for assessing multicollinearity. The results produced tolerance values between 0.57 and 0.72 and VIF values between 1.06 and 1.75, indicating again that multicollinearity was not present in the data (Cohen et al., 2003).

The first hypothesis related to plastic separation (i.e., *H*1) proposed that workers’ perceptions of a green organizational climate are positively associated with their reported waste separation. The results indicate that green organizational climate’s total effect on plastic separation behaviors is significantly positive (*B* = 0.37; *p* < 0.001), so, the stronger employees’ perceptions of a green organizational climate at work, the more likely they are to separate plastic waste. This hypothesis is thus supported.

*H*2 stated that TPB variables mediate the above relationship. The results confirm that green organizational climate has a significant positive association with attitude (*B* = 0.22; *t* = 3.22; *p* < 0.001), subjective norms (*B* = 0.65; *t* = 11.06; *p* < 0.001), and perceived behavioral control (*B* = 0.50; *t* = 11.96; *p* < 0.001). These three factors are also positively and significantly related to plastic recycling as follows: attitude (*B* = 0.13; *t* = 2.26; *p* < 0.05), subjective norms (*B* = 0.17; *t* = 2.65; *p* < 0.05), and perceived behavioral control (*B* = 0.55; *t* = 6.94; *p* < 0.001). In addition, their indirect effect is positive and significant, which provides support for TPB variables’ mediation: attitude [*B* = 0.03; 95% CI (0.29, 0.55)], subjective norms [*B* = 0.11; 95% CI (0.01, 0.23)], and perceived behavioral control [*B* = 0.27; 95% CI (0.18, 0.38)]. The results thus indicate that green organizational climate indirectly affects plastic separation behaviors through TPB variables, in other words, providing empirical support for *H*2. Green organizational climate’s impact is no longer significant in the presence of the mediating variables (*B* = −0.05; n.s.), so complete mediation is present. The resulting model explains 28% of the variation in plastic recycling [*F* (5, 305) = 23.15; *p* < 0.001] (see Table 5).

**Table 5:** Total, direct, and indirect effects for plastic separation.

Variables	Attitude		Subjective norms		Perceived behavioral control		Plastic separation behavior	
	*B*	SE	*B*	SE	*B*	SE	*B*	SE
Total effect								
Constant							2.73^***^	0.26
Green organizational climate							0.37^***^	0.08
Seniority							0.01^*^	0.01
Direct effect								
Constant	5.91^***^	0.23	1.18^***^	0.20	2.06^***^	0.17	0.61	0.42
Green organizational climate	0.22^***^	0.07	0.65^***^	0.06	0.50^***^	0.05	−0.05	0.09
Attitude							0.13^*^	0.06
Subjective norms							0.17^*^	0.07
Behavioral control							0.55^***^	0.08
Seniority	−0.01	0.00	0.00	0.00	0.00	0.00	0.01^*^	0.00
Indirect effect								
	0.03	0.02	0.11^*^	0.05	0.27^*^	0.05		
	95% bootstrap CI							
	[0.00, 0.07]		[0.01, 0.23]		[0.18, 0.38]			

^*^p < 0.05; ^**^p < 0.01; ^***^p < 0.001. *B*, non-standardized regression coefficients; SE, standard error; CI, confidence interval.

The results for *H*3 regarding plastic separation reveal that supervisor support has no moderation effect on any TPB variables as follows: attitude (*B* = −0.04; n.s.), subjective norms (*B* = −0.08; n.s.), and perceived control (*B* = 0.03; n.s.). Therefore, *H*3 is not empirically supported for this type of material (see Table 6). This outcome suggests that supervisors’ support appears to be less important to the relationship between green organizational climate and plastic separation behaviors.

**Table 6:** Moderation and mediated moderation for paper separation.

Variables	Attitude		Subjective norms		Perceived behavioral control	
	*B*	SE	*B*	SE	*B*	SE
Constant	6.64^***^	0.11	3.30^***^	0.09	3.68^***^	0.08
Green organizational climate	0.20^**^	0.08	0.40^***^	0.06	0.50^***^	0.06
Supervisor support	0.03	0.05	0.39^***^	0.04	0.00	0.04
Seniority	−0.01	0.00	0.00	0.00	0.00	0.00
Interaction effect [GreenClimate*SupervisorSupport]	−0.04	0.05	−.08	0.04	0.03	0.04
*R* ^2^	0.04^**^		0.45^***^		0.25^***^	
*F* (4, 306)	3.15		61.52		25.57	
	Moderated mediation index					
	−0.01		−0.01		0.02	
	95% CI					
	[−0.03, 0.01]		[−0.04, 0.00]		[−0.04, 0.07]	

^*^p < 0.05; ^**^p < 0.01; ^***^p < 0.001; B, non-standardized regression coefficient; SE, standard error; CI, confidence interval.

## 5. Discussion

This research focused on corporate environmental sustainability (Magill et al., 2020; Duarte and Mouro, 2022) in order to respond to a specific tertiary sector organization’s request for help with an intervention proposal to increase its level of waste (i.e., paper and plastic) separation in its main building. This study sought to understand the interactions between green organizational climate and perceived supervisor support with individual-level TPB variables in predicting waste separation. More specifically, analyses were conducted to assess these variables’ mediating effect on the relationship between green organizational climate and waste separation, and to determine whether supervisor support moderates the links between green organizational climate and TPB variables.

The results confirm that a green organizational climate is positively associated with the self-reported separation of paper and plastic waste, which corroborates *H*1. By addressing environmental issues and concerns, the organization in question promotes its workers’ adoption of PEBs, as shown by previous literature (Temminck et al., 2015; Mouro and Duarte, 2021). Organizations such as this one can further promote positive perceptions of environmental sustainability among workers and effectively increase PEBs by directly engaging staff members in planning and implementing initiatives in this area (Snider et al., 2003).

The present findings clarify TPB variables’ mediating role in the relationship between green organizational climate and waste separation behaviors as complete mediation was confirmed for both paper and plastic waste, thereby corroborating *H*2. Workers’ perception of a green organizational climate strengthens their attitude, subjective norms, and perceived behavioral control, which in turn are linked with more frequent waste separation in the workplace. This result supports the existing literature by demonstrating that attitude (Russell and Griffiths, 2008), subjective norms (Afsar and Umrani, 2020), and perceived behavioral control (Lülfs and Hahn, 2013) are all associated with green organizational climate, and all three variables are related to higher engagement in waste separation (Norton et al., 2015; Ofstad et al., 2017; Wesselink et al., 2017). Perceived behavioral control is the strongest predictor for both types of residues, not only corroborating previous studies on waste separation (Blok et al., 2015; Botetzagias et al., 2015; Ofstad et al., 2017) but also supporting the TPB assumption that this factor more directly affects engagement in behaviors (Ajzen, 1991).

Regarding *H*3, the current results verify that supervisor support moderates the relationship between green organizational climate and subjective norms for paper separation. The more strongly employees perceive managers as encouraging paper recycling, the weaker the association becomes between green organizational climate and subjective norms. In other words, when supervisor support for environmental issues is believed to be strong, workers’ perception that their organization has a green organizational climate is less closely associated with their perception of social pressures exerted by close colleagues and other employees on them to participate in PEBs. This finding suggests that, because supervisors are closer to their subordinates, they can to some extent eclipse the rest of the organization’s influence, providing their workers with social norms and inspiring the relevant work groups to support environmental sustainability practices more fully at work (Daily et al., 2009).

The results thus indicate that green organizational climate plays a determining role in workers’ separation of waste in the workplace. In addition, TPB variables—attitude, subjective norms, and perceived behavioral control—are mediators of this relationship, thereby contributing to waste recycling. These findings show that organizations can foster or strengthen employees’ closer alignment with environmental concerns. Supervisor support also moderates the connection between green organizational climate and subjective norms for paper separation. This relationship had not previously been analyzed by researchers, so the present results contribute to clarifying supervisors’ role in the transition to sustainability.

### 5.1. Theoretical and practical implications

These above outcomes contribute in different ways to the existing literature on PEBs at work. First, this investigation expanded on recent research by clarifying the links between these behaviors’ background processes. The results are consistent with the view that TPB variables are predictors of waste separation behavior, but, as the extant literature suggests, the three TPB factors have a different weight in terms of predicting specific PEBs (Greaves et al., 2013; Botetzagias et al., 2015; Norton et al., 2015; Razak and Sabri, 2019).

The present findings additionally show that green organizational climate, supervisor support, and subjective norms combine to form an integrated, interrelated conceptual structure supporting workers’ adoption of PEBs. Direct supervisors in daily contact with subordinates are responsible for transmitting and demonstrating their organization’s environmental policies, procedures, and practices, so supervisor support is essential to stimulate employees to become more concerned about environmental problems at work (Paillé et al., 2019).

Regarding practical implications, the current findings indicate that organizations must frequently communicate information about environmental initiatives, activities, and policies to workers. Companies can, for example, organize meetings, workshops, or seminars to promote sustainability-related behaviors and apprise staff members of procedures and functions associated with the waste separation systems implemented. More information also needs to be provided on how to separate paper and plastic correctly. In this context, four aspects are especially important: convenience (i.e., facilitating access to structures and providing reminders), information (i.e., providing reasons for behaviors and ways to participate in them), monitoring (i.e., providing feedback and rewards), and social modeling (Osbaldiston and Schott, 2012). The selection of specific activities to encourage PEBs should be based on specific objectives and the characteristics of the target population (Grilli and Curtis, 2021).

The present study further highlighted different levels of perceived control for plastic and paper residue, suggesting that for plastic it is necessary a stronger focus on clarifying separation conditions than for paper. During interventions, supervisors must also express support for their organization’s more sustainable practices (Jones et al., 2012). The current research’s results indicate that supervisor support is more important for paper recycling, especially as a normative referent.

### 5.2. Limitations and suggestions for future studies

This study has some limitations that should be considered when interpreting the results. The first was the convenience sampling method, which makes generalizing the results to the general population or other organizations problematic. More studies are needed to validate the proposed model in other organizations and business sectors. Second, the research was conducted in partnership with a specific company, which was not authorized to share the participants’ emails, so a random selection of employees was not feasible. The self-selected sample may have introduced bias as workers more concerned about environmental issues may have been more willing to fill out the questionnaire due to its central focus, causing these individuals to be over-represented in the data.

Another possible limitation was the self-assessment items used to measure PEBs because this method is considered by some scholars to be one of the main problems in the existing literature. However, previous research has found that social desirability has a low or zero effect on how people report their PEBs in anonymous questionnaires (Milfont, 2009), and the present study guaranteed the respondents’ anonymity. No personal data were requested, such as name or employee number, that could lead to the identification of individuals. In addition, the participants were informed that the data gathered with the questionnaires would be confidential, the data were intended only for statistical treatment by the research team, and no responses would be analyzed or reported individually. Nonetheless, future investigations may benefit from collecting objective statistics, such as the amount of waste collected in the organization under study in a given period.

The present research was cross-sectional and correlational, so the analyses identified correlations instead of causality links. A longitudinal design could be used in further studies to establish univocal cause and effect associations. Additional research is needed to explore other individual-level variables—for example, empowerment (Paillé and Francoeur, 2022) attitudinal ambivalence (Mouro et al., 2021), and habits (Alzaidi and Iyanna, 2022)—and organization-related variables—such as organizational pride (Raza et al., 2021), person-organization fit (Duarte and Mouro, 2022), green human resource practices (He et al., 2021), and CSR skepticism and authenticity (Latif et al., 2022)—that may further contribute to intervention proposals that promote PEBs in the workplace. Given the importance of supervisor support, future studies could also dedicate more attention to how this specific category of employees develops their own green attitudes and behaviors at work. Recent research has confirmed the significant role of green human resource management and green organizational climate in supervisors’ PEBs (Rubel et al., 2021). More investigations are thus needed of the ways managers can be stimulated to participate in these behaviors in their workplace, including engaging in green advocacy and reinforcing their subordinates’ PEBs.

As mentioned, this research took place just before the first lockdown imposed by the Covid-19 pandemic, with workers developing their work activities on-site at the organization’s main building. With the digitalization of work boosted by the Covid-19 pandemic, the work regimes have changed for these and many other workers with an increasing number of people adopting telework or hybrid work regimes. For these remote or hybrid workers, the ‘workplace’ is no longer or, at least, is not always at the organizations’ premises. This change creates several opportunities for future research. What impact does the work regime has on PEBs at work, i.e., during work activities? How can organizations stimulate PEBs during work activities outside their premises? As the suitability of prior knowledge for these new work settings is unknown, more research is needed on the factors affecting PEBs in non-traditional work settings.

## 6. Conclusion

This study sought to examine how organizations can encourage their workers to join in PEBs in their facilities. The results highlight the importance of investing in internal green policies and practices as part of an overall transition strategy to achieve sustainability. The TPB can be an important tool that companies can use to identify barriers to and facilitators of PEBs at work, as well as more specifically clarify how individual employees’ actions can contribute to their organization’s broader ecological goals. However, individual TPB determinants need to be strengthened in combination with organizational factors because both the organization’s investments and supervisors’ incitement give meaning and structure to workers’ personal environmentally friendly.

## Data availability statement

The raw data supporting the conclusions of this article will be made available by the authors, without undue reservation.

## Ethics statement

Ethical review and approval was not required for the study on human participants in accordance with the local legislation and institutional requirements. The patients/participants provided their written informed consent to participate in this study.

## Author contributions

CM and APD supervised the study’s development. AC, CM, and APD formulated the study’s plan, designed the data collection process, conducted the analyses, and wrote this article. All authors contributed to the article and approved the submitted version.

## Funding

This research was partially supported by Portugal’s Fundação para a Ciência e Tecnologia *via* grants UIDB/00315/2020 and UID/03125/2020, and contracts DL57/2016/CP1359/CT0006 and DL 57/2016/CP1359/CT0004 awarded to CM and APD.

## Conflict of interest

The authors declare that the research was conducted in the absence of any commercial or financial relationships that could be construed as a potential conflict of interest.

## Publisher’s note

All claims expressed in this article are solely those of the authors and do not necessarily represent those of their affiliated organizations, or those of the publisher, the editors and the reviewers. Any product that may be evaluated in this article, or claim that may be made by its manufacturer, is not guaranteed or endorsed by the publisher.

## 8. Appendix

**8.1. Green organizational climate**

My organization…
…is willing to support its workers’ efforts to implement environmentally friendly practices.
…encourages its workers to engage in environmentally friendly practices.
…has an adequate system for separating waste for recycling.
…is available to find new solutions for environmental problems.
…ensures that the cleaning services adequately separate paper and plastic for recycling.
… provides appropriate containers for paper separation.
… provides information on how to separate paper for recycling.
… provides appropriate containers for plastic separation.
… provides information on how to separate plastic for recycling.

**8.2. Waste separation behavior**

I have separated paper/plastic waste for recycling for the last 4 weeks.

**8.3. Attitude**

To separate paper/plastic for recycling is something …
1 = positive; 7 = negative.
1 = useful; 7 = useless.
1 = that makes me feel good; 7 = that does not make me feel good.
1 = that I value; 7 = that I do not value.

**8.4. Subjective norms**

My direct supervisors expect me to separate paper/plastic waste.
My closer coworkers expect me to separate paper/plastic waste.
My colleagues working in this building expect me to separate paper/plastic waste.

**8.5. Perceived behavioral control**

I know where to take the paper/plastic for recycling.
It’s difficult for me to know the conditions in which paper/plastic can be recycled.
I find it inconvenient to separate paper/plastic in my workplace.
I find it easy to separate paper/plastic in my workplace.
To separate paper/plastic in my workplace is something out of my control.

**8.6. Supervisor support**

My direct supervisor…
… encourages me to incorporate environmentally friendly practices into my work.
… gives me helpful advice on environmental practices in the workplace.
…values environmentally friendly behaviors in the workplace.
